# Nocturnal Terrors in Children

**Published:** 1884-09-20

**Authors:** E. Van Goidtsnoven

**Affiliations:** Georgia


### Southern Medical Record:

EDITORS:

THOMAS S. POWELL, M.D. R. C. WORD, M.D.

*R. C. WORD, M.D., Managing Editor.*

All Communications and Letters on Business connected with the RECORD must be addressed to the Managing Editor.

VOL. XIV. ATLANTA, GA., SEPTEMBER 20, 1884. No. 9.

### ORIGINAL AND SELECTED ARTICLES.

### NOCTURNAL TERRORS IN CHILDREN.

Translated for THE SOUTHERN MEDICAL RECORD from the Paris Journal de Medecine,

BY E. VAN GOIDTSNOVEN, M.D., OF GEORGIA.

Dr. Moizard, in the Revue des Maladies de l' Enfance, dwells on the various symptoms and characters of those accidents peculiar to children, and on their respective etiology.

"A child of three to six years," says he, "is quietly sleeping, and nothing abnormal has, thus far, been noticed in his condition, when, on a sudden, after three or four hours rest, he startles and sits on his bed. His eyes are wide open and riveted, so to speak, on a terrifying apparition which he endeavors to avert. Everything about him expresses fright. His features are distorted; he cries out plaintively; utters incoherent words, some of which may lead to the discovery of the true cause and point out the spectral hallucination as the circumstance producing terror. It may be a hideous monster, robbers, etc., etc. The child does not recognize the persons who surround him, and who have quickly come to his relief upon hearing his shrieks. He, nevertheless, throws himself in their arms, as if he were seeking a refuge against the impending danger. Then, all at once, after five minutes, half an hour or an hour of agitation and fright, calm is restored, the child drops again into a tranquil sleep, and, on the morrow, awakes re-comforted and cheerful, rarely remembering the terrifying scene of the night. Such are the principal phenomena which characterize nocturnal terrors. A few symptoms will complete the description. During the crisis the child seems to be out of his mind. He calls for his mother, but does not recognize her voice. No convulsions have ever been observed, either before or after the crisis; and this is a very important point in diagnosis. Ordinarily, there occurs but one attack per night, yet the paroxysm repeats itself for several consecutive nights; and these spells may last a month or six weeks. In a case observed by Dr. Moizard, the crisis lasted six weeks, and began every night at ten o'clock with such a regularity that, wishing to witness some of the paroxysms, the doctor would arrive at the home of the child a few minutes before the recurrence, which was invariable in its punctuality. This was followed by an intervening period of quietude lasting one month. The paroxysms would again recur every night for two weeks, and then from time to time only, whenever the digestive troubles, which evidently were the cause of this disorder, resumed their morbific agency. In the intervals between paroxysms nothing abnormal in the condition of the child ordinarily presents. Babies, however, are generally excitable, and after the cries of terror have ceased, other nervous symptoms, somnambulism for instance, may sometimes be observed.

"Amongst the predisposing causes of this disorder may be cited the nervous and impressionable condition of children and the horrible custom of frightening their imagination with bugbear stories, walking spectres, idle phantoms, violent scenes, etc., etc.

"But there are other causes affecting the majority of cases, and Dr. Debacker, who has written an excellent work on the subject, lays down two distinct orders of causes very important with respect to prognostic and treatment; namely, those which act indirectly on the brain and affect it through reflex action, producing, doubtless, transitory troubles within the circulation, and those which strike directly the encephalon and are in constant relation with a permanent lesion, In the first category we place digestive troubles, which, in the child, are apt to produce nervous phenomena of grave importance, convulsions, aphasia, transient hemiplegia, etc. Over-feeding, abuse of alcoholic medications, may determine those accidents subsequently made durable by resulting dyspepsia. Moreover, it must be remarked that some children are subject to very singular idiosyncrasies. For instance, a certain kind of food universally acknowledged to be very wholesome and digestible may never be ingested into the stomach of some child without producing one of those spells of terror, as above described.

"Constipation, intestinal worms, especially oxyures, some remedies—such as belladonna, datura, and sometimes sulphate of quinine—according to the observations of Dr. Simon, may produce these nocturnal terrors. Although the teething period, in most instances, is over, its baleful influence may, at times, be considered. Finally, persisting irritation of the skin, acne, prurigo, etc., have been, also, mentioned as morbi causæ.

"In the second category of causes we place tuberculous meningitis, cerebral sclerosis, of which nocturnal terrors may be considered as forerunners. Terrors may, also, be a symptomatic manifestation of epilepsy and hysteria

"It is obvious that the prognosis, as well as the treatment of this affection absolutely depend on its cause, which should always be investigated. The preponderating influence of digestive troubles indicate the necessity of a rigid diet. Intellectual hygiene is of no less importance, and any excess of mental work should be strictly avoided. Finally, as children of delicate complexion are often involved, regular exercise and a regime both restorative and tonic are necessary.

"As to the paroxysm itself, bromide of potassium and chloral appear to be the best remedies indicated. It should be remembered that these attacks almost invariably supervene in the first part of the night, and they should, therefore, be guarded against and prevented by securing a sound and quiet sleep during the first three or four hours of the night."

